# Acupuncture for “poor ovarian response” of women to controlled ovarian hyperstimulation: A protocol for meta-analysis and systematic review

**DOI:** 10.1097/MD.0000000000022868

**Published:** 2020-10-30

**Authors:** Wei Wei, Li-Ying Liu, Ling Chen, Meng-Hua Su, Xiao-Juan Hong

**Affiliations:** Acupuncture and Tuina School, Chengdu University of Traditional Chinese Medicine, Chengdu, China.

**Keywords:** acupuncture, meta-analysis, poor ovarian response, protocol, systematic review

## Abstract

**Background:**

Poor ovarian response (POR) is a high-incidence disease of women, which cause in vitro fertilization failure. Various treatment options have been proposed for women with POR to improve their ovarian response, but with little effect. In recent years, there is a wide range of applications of acupuncture in the process of in vitro fertilization. The meta-analysis and systematic review are designed to analyze whether acupuncture is effective for patients with POR.

**Methods:**

The following databases will be searched from inception to March 2020: Electronic databases consist of MEDLINE, EMBASE, Allied and Complementary Medicine Database, China National Knowledge Infrastructure, Chinese Biomedical Literature Database, the Chinese Scientific Journal Database, and Wanfang Database. Other literature resources will also be searched including clinical trial registries, key journals, and meeting records. The results of randomized controlled trials of acupuncture therapy on POR, which are published in Chinese or English, will be embedded. The primary outcome is the clinical pregnancy rate. Data identification, data selection, data extraction, and assessment of bias risk will be completed independently by 2 or more reviewers. STATA/IC 16 will be used to perform the meta-analysis. We will use the Grading of Recommendations Assessment, Development, and Evaluation system to evaluate the quality of our evidence. A systematic narrative synthesis will be provided if the quantitative analysis is not available.

**Discussion:**

This study will provide the first meta-analysis and systematic review to evaluate the efficacy of acupuncture in treating POR. This protocol provides details to guide this study.

**Conclusions:**

From this review may benefit POR patients or clinical decision-makers.

**PROSPERO registration number:**

PROSPERO CRD42020169560.

## 1 Introduction

### 1.1 Description of the condition

Controlled ovarian hyperstimulation (COH) was proposed for recruiting adequate follicles to boost the success rates of in vitro fertilization (IVF). However, there are still “poor responder” who respond poorly to COH resulting in low success rates with IVF.^[1–4]^ Despite rapid development in IVF, poor ovarian response (POR) remains one of the most challenging tasks faced by reproductive clinicians. The prevalence of POR varies from 5.6% to 35.1%^[3]^ and brings the risk of cycle cancellation of 20%.^[5]^ The pathogen is unclear in half of those patients with POR.^[6]^ Varieties of interventions exist to improve pregnancy rate for patients with POR, however, no definitive consensus is accepted for the best treatment.^[7–9]^ In summary, the application of adjuvant therapy to improve IVF results for POR patients should be taken into consideration.

### 1.2 Description of the intervention

Acupuncture, as a unique method in traditional Chinese medicine, involves inserting fine needles and simulating acupoints in specific parts of the body to regulate the meridian for health recovery. Acupuncture therapy has been shown may have effects on infertility.^[10–13]^ However, only 1 systematic review^[14]^ demonstrated that acupuncture might improve clinical pregnancy rate (CPR), anti-Mullerian hormone (AMH), antral follicle count, and the number of retrieved oocytes in POR patients undergoing IVF. In contrast, it is hard to conclude that acupuncture is more effective than traditional therapies for POR. Furthermore, an increasing number of clinical studies showed that acupuncture improves the IVF outcome of POR patients.^[14,15]^

### 1.3 How the intervention might work

There are pathological and physiologic studies on acupuncture in treating infertility. Acupuncture can regulate the function of the hypothalamic-pituitary-ovarian axis by modulating endogenous opioids especially β-endorphin, thereby affecting the release of gonadotropin-releasing hormone.^[16–18]^ Studies have revealed that acupuncture also affected peripheral gonadotropin levels of women including follicle stimulating hormone, luteinizing hormone, estradiol, and progesterone.^[19,20]^ Chen^[18]^ found that electro-acupuncture could normalize the dysfunction of the hypothalamic-pituitary-ovarian axis by altering the expression of some genes in the brain. Studies also revealed that electro-acupuncture could improve uterine and ovarian blood flow of infertile women.^[21,22]^ Acupuncture can facilitate implantation by inhibiting overactive sympathetic nervous systems.^[23,24]^ Poor ovarian reserve is a cause of poor response to COH.^[25]^ Acupuncture showed a benefit of improving the ovarian reserve of patients, while the quality of researches was weak and not representative.^[26–29]^ Further research is needed.

### 1.4 Why it is essential to perform this review

Inducing ovulation in patients with POR during IVF is one of the most challenging but frustrating issues in reproductive medicine. Even though improving IVF outcomes for POR women is a major priority, the lack of a unique definition of POR hinders researches in this area.^[30]^ Despite differences in the definition of POR, the treatment cycle of POR patients often has a meager success rate or is canceled due to presumed adverse clinical outcomes.^[3,4,31]^ This may bring emotional distress to the couples, and also increase the financial burden on them.^[4,31]^ The cost of treatment may vary depending on different treatments, though the live birth rates resulted similarly.^[4,7]^ Normally, follicle donation seems the only option for women with POR. It takes lots of time for couples to wait for the right donor.

Although acupuncture has been used to improve pregnancy rate among women undergoing IVF,^[32–34]^ there is only 1 systematic review to describe the effectiveness of acupuncture in improving the CPR after IVF.^[14]^ We will provide clinicians with more precise data on the effectiveness of acupuncture of POR patients during COH in the context of IVF as well as identify areas where further evaluation is necessary.

### 1.5 Objectives

To compare the effectiveness of different kinds of acupuncture therapies in women with POR during COH in the context of IVF.

## 2 Methods/ design

### 2.1 Eligibility criteria

#### 2.1.1 Types of studies

Randomized controlled trials (published and unpublished) of acupuncture intervention will be included in the review. Languages of all alternative literature shall be Chinese and English. Uncontrolled clinical trials and crossover trials with no first phase data will be excluded. We also remove studies without an identifiable definition of POR.

#### 2.1.2 Types of participants

Participants diagnosed with POR will be included. Diagnostic criteria are not limited. All participants undergo IVF with acupuncture intervention during any time of IVF. There is no restriction on age or ethnicity of the enrolled subjects.

#### 2.1.3 Types of interventions

All acupuncture therapies considered in the study are defined as either traditional acupuncture with needle stimulation of acupoints or modern acupuncture technique without needling. COH therapy of any types should be assessed along with acupuncture therapy in both groups.

#### 2.1.4 Types of control/ comparator(s)

We will classify comparators as follow:

1. Acupuncture plus COH therapy versus COH therapy;
2. Acupuncture plus COH therapy versus noninvasive placebo acupuncture/ sham acupuncture plus COH therapy;
3. Acupuncture plus COH therapy versus herbs plus COH therapy.

We will exclude studies comparing the effects on different types of acupoints or forms of acupuncture.

#### 2.1.5 Types of outcomes

##### 2.1.5.1 Primary outcomes

CPR.

##### 2.1.5.2 Secondary outcomes

1. Other indicators of pregnancy outcome: pregnancy rates, live birth rate, number of oocytes retrieved per woman, number of received high-quality embryos, fertility rate, cancellation rate;
2. Index for reproductive endocrinology: the value of follicle stimulating hormone, luteinizing hormone, estradiol, antral follicle count, AMH; dose and duration of Gn used for COH;
3. Adverse effects in the process: miscarriage rate, ectopic pregnancy rate.

### 2.2 Information sources

Following databases will be conducted by 2 qualified reviewers (MHS and LC). The search will be performed from inception to March 2020:

- Electronic databases: MEDLINE (via PubMed), Excerpta Medica Database (EMBASE), Allied and Complementary Medicine Database, Index to Chinese Periodical Literature including China National Knowledge Infrastructure, Chinese Biomedical Literature Database, the Chinese Scientific Journal Database, and Wanfang Database.
- Clinical trial registries: Menstrual Disorders and Subfertility Group Specialized Register, Cochrane Central Register of Controlled Trials, World Health Organization International Clinical Trials Registry Platform, Chinese Clinical Registry, and Clinical Trials.
- Manual searches of crucial journals (Human Reproduction Update, Human Reproduction, Fertility, and Sterility) and meetings of European Society of Human Reproduction and Embryology and American Society for Reproductive Medicine for relevant articles.

### 2.3 Search strategy

The search strategy uses a combination of subject words and free words. The following searching terms will be searched: “acupunct∗,” “acupress∗,” “acupoint∗,” “electroacupunt∗,” “electro-acupunt∗,” “moxibust∗,” “point injection,” “transcutaneous electri∗ stimulation,” “point embedding,” “poor ovarian response,” “poor ovarian responder,” “POR,” “controlled ovarian hyperstimulation,” “diminished ovarian response,” “reduced ovarian response,” “ovarian stimulation,” “diminished ovarian reserve,” “low response,” “poor responders,” “in vitro fertilization,” “in vitro fertilisation.” MeSH terms searching be performed using the following terms: “Acupuncture,” “Acupuncture Therapy,” “Transcutaneous Electric Nerve Stimulation.”

The detailed search strategy for PubMed is shown in Table 1. Equivalent translations of above terms will be applied to searching in Chinese databases.

**Table 1:**
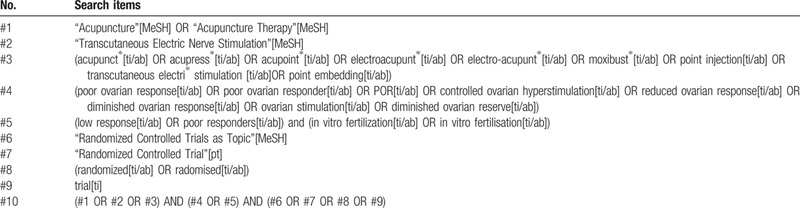
Search strategy of PubMed.

### 2.4 Study selection and data extraction

#### 2.4.1 Data management

LYL is responsible for the management of all documents. Search results will be downloaded by 1 reviewer (LYL) into Microsoft Excel, including citation abstracts and full texts. Our team will formulate screening questions and forms based on inclusion and exclusion criteria in Microsoft Excel. Before the formal screening process, calibration work will be carried out to refine the screening issues. Only research colleagues and other approved people have access to all data of this review. Besides, we will provide training for members who are not familiar with the Microsoft Excel before the review.

#### 2.4.2 Selection process

**Figure 1:**
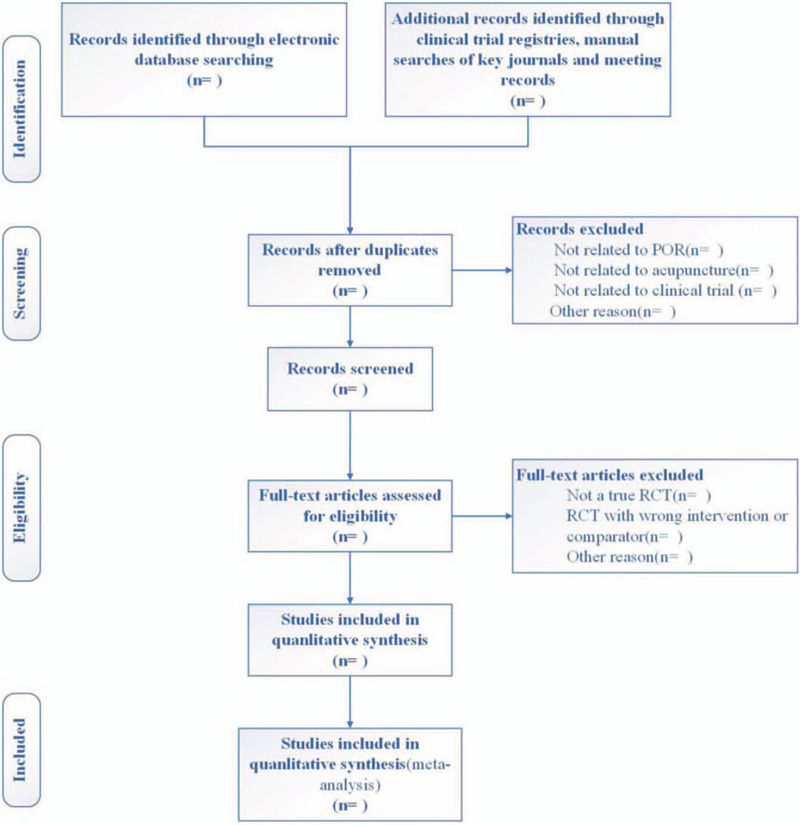
Flowchart of study selection. RCT = randomized controlled trial.

Two independent reviewers (MHS and LC) will respectively screen the obtained articles by reading titles and abstracts with eligibility criteria. Any disagreement between 2 reviewers will be arbitrated by the reviewer (WW) other than the 2. Excluded studies will list the reasons for exclusion in the table of Microsoft Excel. The study selection process will be shown in a PRISMA flow diagram (see Fig. 1).

#### 2.4.3 Data collection process

The following items of included trials will be extracted by 2 reviewers independently (MHS and LC) into a predefined data extraction sheet of Microsoft Excel: general information (article ID author list, year of publication, title, journal name), the methodology of included trials (participant demographics, eligibility criteria, interventions, controls, follow-up measurement), outcomes measurement and so on. Checklist of Standards for Reporting Interventions in Clinical Trials of Acupuncture^[35]^ (STRICTA) is needed to report the details about acupuncture. WW will arbitrate any disagreement existed. The pre-analytical check will be done (MHS and LC) before transfer the data into STATA/IC 16.^[36]^

#### 2.4.4 Dealing with missing data

The reviewers (LYL and WW) will contact the author or relevant authors through phone or e-mail to obtain missing data. If missing data is not available, such studies will be excluded. We will analyze only existing data, and address the potential impact of missing data in discussions.^[37]^

### 2.5 Data synthesis

#### 2.5.1 Meta-analysis

We will use STATA/IC 16 to perform the meta-analysis. For continuous variables, the statistical post-intervention will use Hedges g as the specified effect size. The exact calculation will be used to calculate the bias-correction factor. And the Hedges and Olkin-corrected standard errors will be performed to calculate the effect size. For categorical variables, the specified effect size uses the log Peto ratio. The meta-analysis will use either random-effects model or fixed-effects model. We will assess the DerSimonian-Laird method to perform the random-effects model, while assessing inverse-variance method to perform the fixed effects model.^[38]^ The confidence interval for the meta-analysis is 95%.

#### 2.5.2 Assessment of heterogeneity

*I*^2^ test^[39]^ will be assessed for quantifying between-study heterogeneity. If the *I*^2^ index is less than 50%, the fixed effects model will be performed for analysis. If there is considerable heterogeneity (*I*^2^ index > 50%), subgroup analysis and meta-regression will be performed by the random-effects model.^[38]^

#### 2.5.3 Subgroup analysis

Subgroup analysis will be conducted to interpret the heterogeneity as well. If the number of studies is sufficient, we will conduct subgroup analysis based on the quality of the study, region, age, gender, diagnostic criteria, different types of acupuncture technique, different control interventions.

#### 2.5.4 Sensitivity analysis

We plan to conduct sensitivity analysis if possible after subgroup analysis. Low quality studies will be excluded, then the meta-analysis will be repeated. Decision nodes such as sample size, methodological defects, missing data based on STRITA, and so on.

#### 2.5.5 Systematic synthesis

A systematic narrative synthesis will be provided to summarize and interpret features and results of included studies. A narrative synthesis will explore the relationship and findings within and between studies.

#### 2.5.6 Meta-regression

Meta-regression will be conducted if there is significant heterogeneity. Reviewers will use meta-regression to explore the relationship between acupuncture therapy and the CPR. The following factors will be used as covariates.

References characteristics:

- Year of publication
- Language of citation

Intervention characteristics:

- Duration of acupuncture therapy
- Dose of gonadotropin used for COH

Participant characteristics:

- Mean age
- Ovarian reserve: Value of AMH and AFC

### 2.6 Assessment of risk of bias

The risk of bias will be analyzed using the Grading of Recommendations Assessment, Development, and Evaluation^[40]^ by 2 independent reviewers (LYL and WW). The quality of evidence is classified as “high,” “moderate,” “low” or “very low.”

## 3 Discussion

Acupuncture is recommended to be an effective therapy for infertility in traditional Chinese medicine. This study has potentials to provide evidence to identify whether acupuncture is effective for treating POR in patients undergoing IVF. Based on the previous research,^[14]^ we will improve our study by discussing the improvement of AMH in acupuncture treatment for POR patients, increase the number of search databases. If heterogeneity deems it possible, we will provide detailed subgroup analysis and meta-regression analysis. Conclusions from this review may benefit POR patients or clinical decision-makers. We will update the protocol if necessary, as well as explaining changes and providing the date of each modification as supplements.

## Author contributions

**Writing – original draft:** Wei Wei, Li-Ying Liu, Ling Chen, Meng-Hua Su.

**Writing – review & editing:** Xiao-Juan Hong.
